# Exploiting Non-Conventional Yeasts for Low-Alcohol Beer Production

**DOI:** 10.3390/microorganisms11020316

**Published:** 2023-01-26

**Authors:** João Simões, Eduardo Coelho, Paulo Magalhães, Tiago Brandão, Pedro Rodrigues, José António Teixeira, Lucília Domingues

**Affiliations:** 1 CEB–Centre of Biological Engineering, University of Minho, 4710-057 Braga, Portugal; 2 LABBELS—Associate Laboratory, Braga, 4835-198 Guimarães, Portugal; 3 Super Bock Group, SGPS, SA, 4466-955 Leça do Balio, Portugal

**Keywords:** non-conventional yeast, fermentation, beer, brewing potential

## Abstract

Non-*Saccharomyces* yeasts represent a very appealing alternative to producing beers with zero or low ethanol content. The current study explores the potential of seven non-*Saccharomyces* yeasts to produce low-alcohol or non-alcoholic beer, in addition to engineered/selected *Saccharomyces* yeasts for low-alcohol production. The yeasts were first screened for their sugar consumption and ethanol production profiles, leading to the selection of strains with absent or inefficient maltose consumption and consequently with low-to-null ethanol production. The selected yeasts were then used in larger-scale fermentations for volatile and sensory evaluation. Overall, the yeasts produced beers with ethanol concentrations below 1.2% in which fusel alcohols and esters were also detected, making them eligible to produce low-alcohol beers. Among the lager beers produced in this study, beers produced using *Saccharomyces* yeast demonstrated a higher acceptance by taster panelists. This study demonstrates the suitability of non-conventional yeasts for producing low-alcohol or non-alcoholic beers and opens perspectives for the development of non-conventional beers.

## 1. Introduction

Beer is one of the most popular beverages worldwide. Traditionally, it is produced from brewed malt, hops, and water, and is fermented with yeast [1]. It is characterized by pleasant organoleptic attributes and with favorable nutritional characteristics when consumed at light-to-moderate levels, such as B vitamins, minerals, and antioxidants, [1,2]. Although the beer industry’s market growth is slowing down, interest in non-alcoholic and low-alcohol beer (NABLAB) is increasing, reflecting a society that is more concerned about health and well-being [3]. 

However, NABLABs cope with organoleptic concerns as their production practices compromise flavor, reducing a broader consumer acceptance [1]. The production of NABLABs can be accomplished by removing ethanol from a standard alcoholic beer (de-alcoholization) or by biological methods which limit the formation of ethanol during fermentation [3]. In both methods, aroma compounds are negatively impacted or are lacking due to their removal along with the ethanol fraction in the first method or the low-to- null formation in the second method [4]. 

The biological approach can be separated into two categories: methods that can be used with standard brewing equipment and methods that require special equipment [3]. Alterations in mashing, arresting, or limiting fermentation and the use of non-conventional yeast can be accomplished with standard brewery equipment [5], whereas continuous fermentation involves investment in specific equipment [6].

The study of non-conventional yeasts with a limited capability to ferment wort sugars has gained increasing research interest. Wort-sugar composition is characterized by high contents of maltose and maltotriose, with lower contents of glucose, fructose, and sucrose. Thus, the selection of yeast unable to ferment maltose and maltotriose results in a lower production of ethanol, as it only ferments the remaining sugars available in minor concentrations [4]. 

A yeast that is suitable to produce a NABLAB should have several characteristics, namely: be unable to ferment maltose; be able to grow in the presence of hops iso-α-acids, does not produce phenolic off-flavors (POF), can easily flocculate, and is safe for human consumption [7,8].

These non-conventional yeasts can be found and isolated from several different sources, which include: brewery contamination yeasts (*Trigonopsis cantarellii* and *Candida sojae*) [9], grape must (*Saccharomycodes ludwigii*) [10], cold habitats, such as Antarctica (*Mrakia gelida*) [8], honey (*Zygosaccharomyces rouxii*) [10], fermented honey by-products (*Hanseniaspora uvarum, Wickerhamomyces anomalus, Z. rouxii,* and *Zygosaccharomyces bailii*) [11], kombucha (*Hanseniaspora vineae*, *Hanseniaspora valbyensis*, *Torulaspora delbrueckii*, *Zygosaccharomyces kombuchaensis*, and *Z. bailii*) [7,12], masau fruit (*Cyberlindnera fabianii, Pichia kudriavzevii*) [13], traditional chicha beverages (Andean beer) (non-conventional *Saccharomyces cerevisiae* ERS1 and EYS4 strain, and *Torulaspora delbrueckii*) [14,15], pickle (*Z. rouxii*) [10], sourdough cultures (*Kazachstania servazzii* and *Pichia fermentans*) [16], wine and sake fermentations (*Lindnera mrakii*) [17], various exotic plants (*Hanseniaspora uvarum*, *Pichia kluyveri*, *S. cerevisiae* Gr112, and *Hanseniaspora guilliermondii*) [18], and from other industrial applications such as biofuel production (*Candida shehatae*) [19].

Several yeasts have been studied in an effort to produce non-alcoholic beer with promising results, such as *S. ludwigii*, which was described as unable to catabolize maltose and with low ethanol production. De Francesco and colleagues used *S. ludwigii* on a small scale of 12°P wort at 20 °C under aerobic conditions and obtained ethanol concentrations ranging from 0.51 to 1.36% (*v/v*) [10]. Furthermore, yeasts from the *Pichia* genus were described as producers of low levels of ethanol when grown in wort, producing a concentration of 0.1–0.7% (*v/v*). Liu and Quek inoculated *L. mrakii* in a wort with 13.8°P and incubated it at 21 °C for fourteen days, producing a beer with an ethanol content of 1.7% [17]. Bellut and colleagues characterized yeasts from the genus *Hanseniaspora* and observed that they only fermented glucose and fructose (without using sucrose, maltose, or maltotriose) [7]. Moreover, in a fermentation trial on 1.5 L of 6.6°P wort at 25 °C, it achieved final ethanol contents of 0.34–0.35% (*v/v*). Another promising yeast is *Z. rouxii*, which demonstrated an ethanol production of 0.93% (*v/v*) and positive aromatic characteristics [10].

This work presents a comparative study of the brewing potential of non-conventional species to produce a NABLAB at a low temperature (14 °C). Species were chosen based on being previously described as low-ethanol producers and were preferably isolated from beer. The selected yeast species were: *H. valbyensis, P. fermentans, L. mrakii, S. ludwigii, Z. rouxii*, and *Candida spandovensis.* Additionally, two commercially available yeasts and two new *Saccharomyces cerevisiae* strains from Reinassance (Lallemand), created by selective breeding and adaptive evolution, designated as MN-229 and MN-851, were investigated. From the initial pool, eight strains were further studied in two L-scale wort fermentations, after which chemical and sensory analyses were performed on the resulting beers. The present study highlights how yeast strains and traditional equipment can be used for NABLAB and non-conventional beer production.

## 2. Material and Methods

### 2.1. Yeast Strains and Media

The yeast strains used in this study were obtained from NCYC, National Collection of Yeast Cultures, (Quadram Institute Bioscience, Norwich Research Park, Norwich, UK), commercially acquired, or gently provided by Renaissance BioScience, Vancouver, Canada.

The yeast strains were grown in YPD (Yeast extract 1%, Peptone 2%, and D-Glucose 2%), or in standard hopped wort (12°P) from Super Bock Group (Via Norte Aptd. 1044, 4466-955 Leça do Balio). The stocks were kept in glycerol at −80 °C. Table 1 lists the yeast strains that were used in this study.

### 2.2. Initial Screening for NABLAB Production

Pre-inocula were grown overnight in 10 mL of YPD in 50 mL flasks. The strains were washed and re-suspended in a 0.9% NaCl solution and diluted to an optical density (600 nm) of 2. These strains were inoculated into beer wort with a specific gravity of 12°P at a final optical density of 0.1. Fermentations were run in triplicates and conducted in a BioLector (m2p labs) using 48-well round-well plates, with 1 mL of wort per well. Growth was performed at 25 °C and 400 rpm with a controlled humidity of 85%. The yeasts’ growth was monitored by measurement of backlight scattering throughout the fermentation, using a 620 nm scattered light filter with measurement intervals of 20 min.

### 2.3. EBC-Tubes Beer Fermentation

The aerobic propagation of yeast was initiated in duplicate from a single colony on a YPD agar plate to 10 mL of YPD. After incubation at 25 °C for 24 h, the yeast suspensions were transferred to 20 mL of YPD in 100 mL Erlenmeyer flasks with agitation at 200 rpm. Cells were harvested by centrifugation (3000× *g*, 5 min, 20 °C) and inoculated at a concentration of 1 × 10^6^ cells/mL in 2 L of wort with a specific gravity of 12°P. Fermentations were carried out in biological duplicates in 2 L European Brewing Convention (EBC) tubes at 14 °C for 10–12 days, which corresponded to a total consumption of glucose by *S. cerevisiae* strains. To monitor the fermentation, samples of culture were collected aseptically every two to three days.

### 2.4. Wort and Beer Analyses 

#### 2.4.1. HPLC Analysis

Samples from BioLector fermentations were analyzed for ethanol, glycerol, glucose, fructose, and maltose, whereas samples collected from the EBC-Tubes were analyzed for glucose, fructose, sucrose, maltotriose, and maltose. Quantification was performed through high-performance liquid chromatography (HPLC), using a Jasco chromatograph equipped with a refractive index detector (KNAUER k-2300) and an Aminex HPX-87H BioRad column (300 mm × 7.8 mm) maintained at 60 °C. A 5 mmol L^−1^ H_2_SO_4_ aqueous solution was used as the mobile phase at a constant flow of 0.6 mL min^−1^ with 20 µL of sample injection. Quantification was achieved using calibration curves prepared from pure standards; chromatograms were analyzed with the Star Varian chromatography workstation Version 6.3.

#### 2.4.2. Determination of Alcohols, Total Esters, and Total Aldehydes by GC-FID

The determination of n-propanol, isobutanol, isoamyl alcohol, ethyl acetate, isoamyl acetate, diacetyl, 2,3-pentanedione, acetaldehyde, dimethyl sulfide, and ethanol in the beer was carried out in a gas chromatograph with a flame ionization detector GC-FID. Analysis was performed in a gas chromatograph (Varian Star 3400) equipped with a capillary column Supelcowax 10 (60 m, 0.53 mm, Sigma-Aldrich/Supelco) and a flame ionization detector (FID). The temperature was raised from 75 to 130 °C at a rate of 25 °C·min^−1^, with initial and final holds of 11 minutes and 4.5 minutes, respectively. The detector temperature was 250 °C, while the injector temperature was 110 °C. The chromatograph used nitrogen as a carrier gas, with a flow rate of 3 mL·min^−1^. The split ratio was 8:1. The internal standard used was 1,3-dichloropropane. 

#### 2.4.3. Alcolyzer Plus

The pH from the samples collected from EBC-Tubes was evaluated with an Alcolyzer Plus (Anton Paar, Graz, Austria). A densitometer (DMA 4500, Anton Paar) was used for determining the specific gravity of the samples. 

#### 2.4.4. Sensory Analysis

Beers prepared on a 2 L scale were subjected to an organoleptic evaluation on a 0–9 scale, where 0 corresponded to a very low intensity perception and 9 to a very high intensity perception. Panelists evaluated the following characteristics: overall flavor intensity, estery/fruity, floral, worty, malty, cereal-like, Diacetyl, sweetness, sour/acid, bitter, body, and lingering aftertaste. Beers were judged by a group of five panelists with at least five years’ experience in tasting alcoholic beverages (five males, 30 to 50 years old). Beer samples were given in coded, 200 mL tasting cups at a temperature of 8 °C. A radar chart was elaborated, showing the average values of the different attributes for each beer. 

#### 2.4.5. Statistical Analysis

The sensory analysis data was tested following a one-way ANOVA using a Tukey post-hoc test with a significance level of *p* < 0.05, which was carried out to detect significant differences between the variables analyzed depending on the yeast strain inoculated. To study the correlation between each beer characteristic, a Spearman correlation test was performed; it had a significance level of *p* < 0.05. For the principal component analysis (PCA), all beer-tested characteristics were normalized by dividing each value by the sum of all values for that tested characteristic. 

## 3. Results and Discussion

To evaluate the potential of non-conventional yeasts to produce low-alcohol or alcohol-free beer, several characteristics must be verified. One of the key characteristics is the capacity to consume wort sugars. Concerning all-malt beers, the most abundantly present fermentable sugar in wort is maltose (65%), followed by maltotriose (17.5%), glucose and fructose (12%), and sucrose (5%). Subsequently, to produce alcohol-free beers, the yeast should not be able to ferment maltose [7]. 

**Figure 1:**
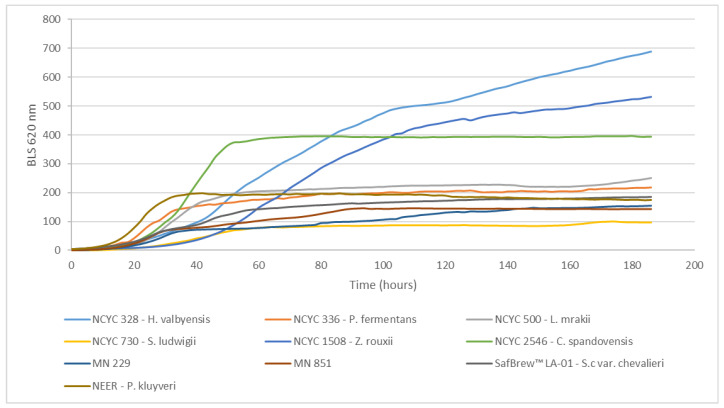
Growth curves of strains carried out in BioLector. The growth of the strains was monitored throughout time (*t*) in a 48-well plate by measuring backlight scatter (BLS) at 620 nm.

All tested yeasts were able to grow in wort (Figure 1). After 186 h, *H. valbyensis* showed the highest value for backlight scatter, followed by *Z. rouxii,* with 77% of the value showed by *H. valbyensis*, followed by *C. spandovensis,* with a backlight-scatter value of 50%. The remaining yeasts showed backlight-scatter values between 14% and 36%. 

As is presented in Table 2, results from the screening showed that all the investigated yeasts were able to ferment glucose and fructose. Concerning maltose, *H. valbyensis* was able to completely consume it and *Z. rouxii* was able to ferment 81%, whereas *P. fermentans*, *L. mrakii*, *S. ludwigii*, *C. spandovensis*, MN-229, and MN-851 were incapable of metabolizing this sugar, showing no substantial differences in concentration at the end of fermentation when compared with wort. This ability/inability to ferment maltose is related to the existence/absence of a functional maltose transporter and the enzyme maltase [20]. The capacity to consume maltose by *H. valbyensis* and *Z. rouxii* supports the higher backlight-scatter value observed in the BioLector fermentations. As these yeasts were able to produce ethanol above 1.2 gL^−1^, they were excluded from further investigation in the production of NABLAB by a biological approach. However, considering the increasing popularity of non-*Saccharomyces* in the beverage industry, these yeasts could prove to be interesting in conjugation with a physical approach for the removal of ethanol in the production of NABLABs.

### 3.1. EBC Tall Tubes

The yeasts that were unable to ferment maltose were selected for further characterization in the EBC-2 L tubes. As was previously observed, all yeasts were able to ferment fructose and glucose. Sucrose fermentation was observed for all strains except for *P. fermentans, L. mrakii,* and the commercial Neer *P. kluyveri* (Table 3). This characteristic can be linked to the absence of the enzyme β-fructosidase (invertase), which hydrolyzes sucrose into glucose and fructose [21,22].

**Figure 2:**
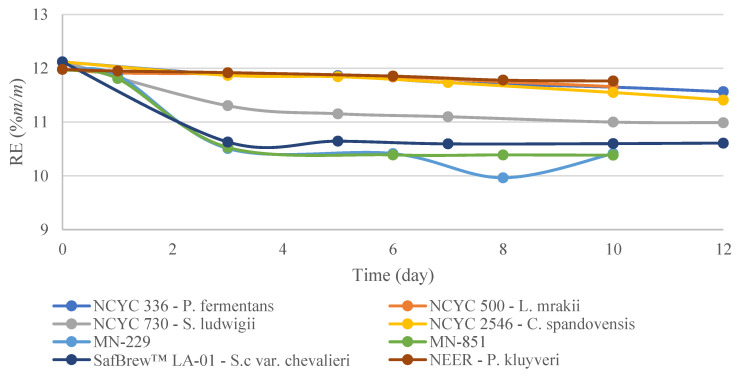
Real extract (RE), drop-in extract for the investigated maltose-negative yeast strains. Results are reported as the mean of two biological replicates.

Wort was fermented between 10 and 12 days, during which *P. fermentans*, *L. mrakii, C. spandovensis*, and the commercial Neer *P. kluyveri* led to a slow decrease in specific gravity and real extract (RE) (Figure 2). Overall, these yeasts caused a drop in extract of approximately 0.7% (*m/m*). *S. ludwigii* and LA-01 *S. cerevisiae* showed a decrease of nearly 1.1% (*m/m*) and 1.5% (*m/m*), respectively. Additionally, *S. cerevisiae*-derived strains MN-229 and MN-851 showed a decrease of 1.5% (*m/m*). 

**Figure 3:**
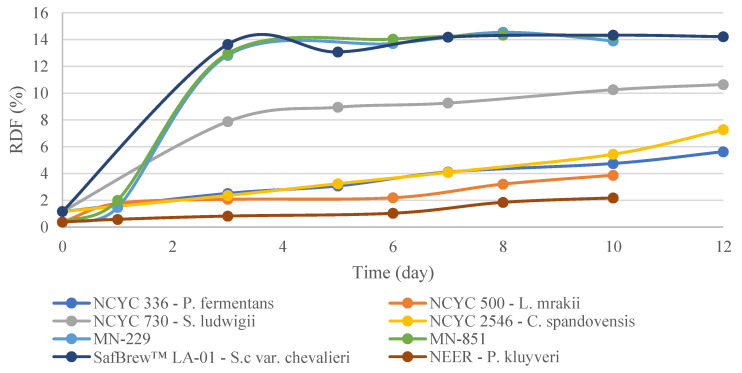
Real degree of fermentation (RDF) content was measured over 10–12 days on the Anton Paar Alcolyzer Beer, as is reported for the fermentation of all yeasts in this study. The results are reported as the mean of two biological replicates.

**Figure 4:**
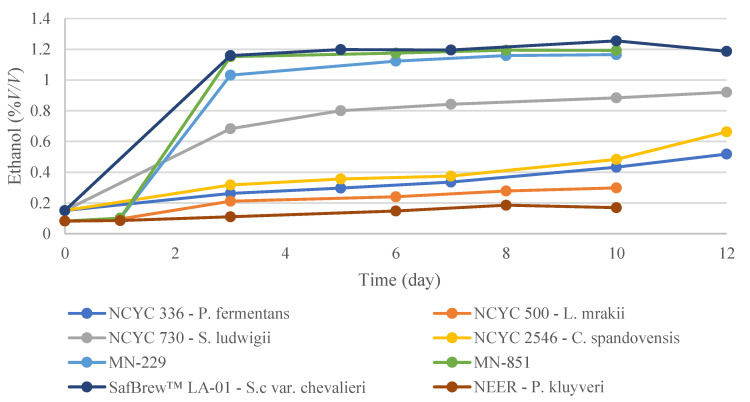
Ethanol content by volume was measured over 10–12 days on the GC, as is reported for the fermentation of all yeasts in this study. Results are reported as the mean of two biological replicates.

Regarding the wort real degree of fermentation (RDF), which reflects the sugar percentage that has been fermented into alcohol, the yeasts that showed the biggest increase were the ones that lowered the extract the most (Figure 3). At the end of fermentation, *S. cerevisiae*-derived strains showed the highest attenuation (MN-851 14.27%; LA-01 *S. cerevisiae* 14.22%; MN-229 13.90%), followed by *S. ludwigii* (10.65%), *C. spandovensis* (7.27%), *P. fermentans* (5.63%), *L. mrakii* (4.75%), and Neer *P. kluyveri* (2.18%). Consequently, the ethanol concentration showed similar behavior to attenuation; at the end of fermentation, LA-01 *S. cerevisiae* and MN-851 showed higher ethanol concentrations, with 1.19 (% *v/v*), followed by MN-229 (1.17% *v/v*), *S. ludwigii* (0.92% *v/v*), *C. spandovensis* (0.66% *v/v*), *P. fermentans* (0.52% *v/v*), *L. mrakii* (0.30% *v/v*), and Neer *P. kluyveri* (0.17% *v/v*) (Figure 4). These results are in line with fructose, glucose, and sucrose consumption (Table 3), whereas the yeasts that fermented the most sugars demonstrated a higher ethanol production.

Concerning pH, the initial wort pH was 4.90 (Figure S1), and this value dropped faster for the yeasts that consumed more sugar and produced the most ethanol, except for the *C. spandovensis* strain, which provided the lowest pH value (4.35) and only produced an ethanol concentration of 0.6% (*v/v*). The remaining yeasts followed the trend that the higher the ethanol production, the lower the pH. LA-01 *S. cerevisiae* showed a decrease in pH to 4.43, followed by MN-229 (4.44), MN-851 (4.47), *S. ludwigii* (4.48), *P. fermentans* (4.60), *L. mrakii,*(4.62), and Neer *P. kluyveri* (4.65) (Figure S1). This pH drop is caused by the increase in yeast population that occurs in the first 2–3 days (Figure 1) and the subsequent ethanol and CO_2_ production, in addition to the consumption of the nitrogen compounds present in wort [23].

### 3.2. Volatile Compounds 

Apart from ethanol, fermenting yeasts also produce an extensive list of secondary metabolites. Although found at much lower concentrations, they are considered the main contributors to the complex aromas of fermented beverages [24]. In this study, several volatile compound groups were analyzed: namely, higher alcohols, esters, vicinal diketones, aldehydes, and sulfur compounds.

Higher alcohols, also known as fusel alcohols, are the most abundant organoleptic compounds present in beer and are generally preferable at concentrations below 300 mg L^-1^, adding complexity and conferring refreshing, fruity, and floral notes [25,26]. Higher alcohols are synthesized by yeast during fermentation, either by catabolism or amino acid metabolism, and can be classified into aromatic and aliphatic alcohols [27]. The principal aromatic alcohols are 2-phenylethanol, tyrosol, and tryptophol; and the principal aliphatic alcohols are n-propanol, isobutanol, and 2-methylbutanol (amyl alcohol) [1,27]. 

Concerning the beers produced, the concentration of higher alcohols (Table 4) was substantially higher for the highest ethanol producers, MN-851 (57.28 mg L^−1^) and *S. ludwigii* (33.47 mg L^−1^), followed by LA-01 *S. cerevisiae* (29.05 mg L^−1^). Propanol and isobutanol did not show any trend; propanol was more concentrated in MN-851 (9.90 mg L^−1^), and less concentrated in *L. mrakii* (2.34 mg L^−1^), with concentrations below the flavor threshold of 600 mg L^−1^ to 800 mg L^−1^ [24,28,29]. Isobutanol was more produced in MN-851(11.32 mg L^−1^) and less in *L. mrakii* (3.16 mg L^−1^), with all concentrations below the flavor thresholds of 100 mg L^−1^ to 200 mg L^−1^ [24,28,29]. Amyl alcohol showed a higher content in MN-851 (36.06 mg L^−1^), followed by *S. ludwigii* (20.87 mg L^−1^) and LA-01 *S. cerevisiae* (20.45 mg L^−1^). In all cases, concentrations were below the flavor threshold of 50–70 mg L^−1^ [30]. The concentration of higher alcohols is dependent on the efficiency of the corresponding amino acid uptake and sugar utilization [31]. The current results agree with previous observations, with the yeasts that consumed glucose faster showing higher concentrations of higher alcohols.

The composition and concentration of fusel alcohols are influenced by the wort composition and yeast-fermentation conditions [27]. Yeast growth and the subsequent production of higher alcohols is promoted by high levels of nutrients such as amino acids, lipids, zinc, and oxygen, as well as agitation and increased temperatures. On the contrary, conditions that decrease yeast growth, such as a low temperature and high carbon dioxide pressure, lead to a decrease in higher alcohol [1,27]. In beer, higher alcohols are involved in several organoleptic characteristics. Aliphatic alcohols such as n-propanol, iso-butanol, and isoamyl alcohols are the main alcohols responsible for the “alcoholic” or “solvent” aroma of beer; they produce a warm mouthfeel [27,31]. Amyl alcohols (2- and 3-methylbutanol) are responsible for “fruity” flavors, whereas N-propanol and 2-methylpropanol may add harsh flavors to beer [32]. Furthermore, isobutanol has an undesirable effect on beer quality when its concentration exceeds 20% of the total concentration of alcohols: n-propanol, isobutanol, and amyl alcohol [33].

Esters are a large group of compounds that confer a fruity flavor aroma. They can have a low aroma threshold and can be subdivided into medium-chain fatty acid ethyl esters and acetate esters [27,31,32,34]. The medium-chain fatty acid ethyl esters include several compounds, such as ethyl caproate and ethyl caprylate (apple flavor) [31,34].

On the other hand, the acetate esters include, among others, ethyl acetate (fruity, solvent-like), isoamyl acetate (banana), and phenylethyl acetate (roses, honey, and apple). Notably, ethyl acetate constitutes approximately one-third of all esters in beers, followed by isoamyl acetate [32]. Ester production by an alcohol–acid reaction occurs in yeast fermentation as a CoA-mediated reaction; subsequently, the availability of the two substrates (alcohols and acetyl/acyl-CoA) and the activity of enzymes (principally alcohol acyltransferases) involved in the formation of esters are factors of crucial importance for the ester formation [31,35].

Ethyl acetate and isoamyl acetate are considered the most important esters for beer flavor because they have relatively low aroma thresholds and frequently occur in concentrations that influence the flavor [36]. Ester (Table 4) concentrations were higher for yeasts that produced less ethanol. Regarding ethyl acetate, *L. mrakii* and Neer *P. kluyveri* exhibited a concentration of 52.18 mg L^−1^ and 32.59 mg L^−1^, respectively: values above the flavor threshold detection of 25–30 mg L^−1^ [30]. The remaining yeasts showed a concentration between 0.665 and 5.56 mg L^−1^. The significantly higher amount of ethyl acetate produced by *L. mrakii* agrees with the observations previously described by Liu and Quek, in which wort inoculated with *L. mrakii* and fermented at 21 °C produced concentrations of ethyl acetate ten times higher than the beer produced with *S. cerevisiae* [17]. With a concentration of 9.06 mg L^−1^, isoamyl acetate was significantly higher in Neer *P. kluyveri*. Above the organoleptic detection threshold of 1.2 mg/L to 2.0 mg L^−1^, the remaining yeasts showed a maximal concentration of 0.935 mg L^−1^ [24,30,37] 

Several additional factors influence ester production: namely, temperature, the presence of oxygen in the wort, pH, amino acid concentration, and the CO_2_ concentration or its pressure inside the fermenter [35]. Furthermore, the presence of different esters can have a synergistic result on the individual flavors, which indicates that esters can impact beer flavor below their individual threshold concentrations [38]. Esters are residual volatile compounds in beer; however, they are important for the flavor profile, being desirable at low concentrations and undesirable at high concentrations [39,40].

The beer content in total higher alcohols and total esters and their ratio is an important indicator in assessing beer flavor. The ideal ratio between higher alcohols to esters for alcoholic lagers is from 4:1 to 4.7:1 [32]. None of the produced beers showed ratio values within the ideal interval: *L. mrakii* and Neer *P. kluyveri* showed a ratio below, 0.3 and 0.4, respectively. Beers that showed better results were from MN-229 (6.6:1), followed by LA-01 *S. cerevisiae* (9.2:1), and MN-851 (9.2:1). The remaining beers showed ratios of 11.1:1 (*S. ludwigii*), 11.5:1 (*C. spandovensis*), and 33.2:1 (*P. fermentans*). These higher ratios cause a dry taste and a less aromatic beer.

Vicinal diketones (VDKs) are flavor compounds present in beverages, most notably diacetyl (2,3-butanedione) and 2,3-pentanedione. They are characterized by aromas and tastes described as “butterscotch”, “honey”, or “toffee”. VDKs are produced as by-products of some amino acid metabolism during fermentation: diacetyl is produced from α-acetolactate, an intermediate in valine and leucine biosynthesis, and 2,3-pentanedione is produced by yeast from the intermediates of isoleucine synthesis [27,36]. These acetohydroxy acid precursors of VDKs are excreted during fermentation and are spontaneously decarboxylated, forming diacetyl and 2,3-pentanedione. These chemical reactions are accelerated by a higher temperature and lower pH [41].

Diacetyl (2,3-butanedione) is more flavor-active, with a flavor threshold of approximately 0.15 mg L^−1^ in lager beer; this is approximately ten times lower than that of pentanedione [27,42]. Lager beers with VDK concentrations above the flavor threshold typically exhibit an unpleasant taste, such as “cheese-like” or sharp [32]. Yeasts have the enzymes necessary to reduce 2,3-pentanedione to 2,3-pentanediol, as well as those required to reduce diacetyl to acetoin and then to 2,3-butanediol [43]. This conversion of diacetyl and 2,3-pentanedione occurs at the end of the main fermentation and during the maturation of beer. Furthermore, the reduced compounds show relatively higher flavor thresholds, and their presence is acceptable at the concentrations typically found in beer [36].

Vicinal diketones (Table 4) were higher in *C. spadovensis*, showing a diacetyl concentration of 0.31 mg L^-1^ and a 2,3-pentanedione concentration of 0.08 mg L^−1^. The other yeasts showed a concentration of diacetyl between 0.07 and 0.14 mg L^−1^, and for 2,3-pentanedione the concentration ranged between 0.015 and 0.029 mg L^−1^. Only *C. spadovensis* showed a diacetyl concentration above the flavor threshold of 0.1–0.15 mg L^−1^ [44]. None of the produced beers showed a content of 2,3-pentanedione above flavor threshold of 1–1.5 mg L^−1^ [44]. These quantifications were made at the end of fermentation; therefore, these values could decrease during beer maturation.

Aldehydes belong to a group of carbonyl compounds that significantly influence the flavor of beer, aldehyde production in beer occurs mainly during wort mashing and boiling, and partially during fermentation from the yeast’s oxo-acid pool via the anabolic process and from exogenous amino acids via the catabolic pathway [1,31,45]. 

Aldehydes have a significantly lower flavor threshold than their corresponding alcohols. The great majority have unpleasant flavors and aromas and, depending on the compound, can be described as “fruity”, “grassy”, “green leaves”, and “cardboard” [1]. Acetaldehyde was the only aldehyde analyzed, as this metabolite represents approximately 60% of the total aldehydes present in beer (Guido et al., 2008). MN-851 showed the highest concentration (30.18 mg L^−1^), followed by MN-229 (25.83 mg L^−1^), *L. mrakii* (20.1 mg L^−1^), Neer *P. fermentans* (15.45 mg L^-1^), *S. ludwigii* (13.29 mg L^−1^), *C. spandovensis* (12.34 mg L^−1^), and *P. fermentans* (10.80 mg L^−1^Finally, there was a significantly lower concentration in LA-01 *S. cerevisiae* (1.47 mg L^−1^). The majority of the tested beers showed a content of acetaldehyde near the flavor threshold of 10–25 mg L^−1^ and, in the concentration range that this metabolite is normally detected in beer, 1 to 20 mg L^−1^ [24,46,47,48,49]. Higher content of acetaldehyde results in “young” or “green” off-tastes, which additionally contribute along phenolics to the formation of beer haze [50].

Sulfur-containing compounds present in beer are formed from raw materials, malt, and hops; however, several are produced by yeast metabolism [36]. Sulfur compounds are essential for yeasts, playing a crucial role in the formation of amino acids, proteins, and coenzyme A. The most important sulfur compounds that can influence beer flavor are hydrogen sulfide (rotten egg: threshold, 8 µg L^−1^), sulfite (pungent: threshold, 10 mg L^−1^), and dimethyl sulfide (cooked cabbage: threshold, 25–50 µg L^−1^) [37,51]. In this work, only dimethyl sulfide (DMS) was monitored (Table 4). This metabolite was more concentrated for the yeasts that produced less ethanol, namely, *L. mrakii* (20.3 mg L^−1^), followed by Neer *P. fermentans* (16.55 mg L^−1^) and MN-229 (16.59 mg L^−1^). The other yeasts showed a concentration between 6.85 and 11.95 mg L^−1^. All these concentrations are above the flavor threshold. These compounds are produced from the sulfate, sulfite, and sulfide ions that are present in wort [52]. Hydrogen sulfide and sulfur dioxide are produced by the yeast during fermentation and are important for the biosynthesis of sulfur-containing amino acids such as methionine and cysteine; subsequently, these amino acids are involved in the aromatic structure of beer [52,53]. 

On the other hand, dimethyl sulfide (DMS), can be produced by the thermal degradation of S-methylmethionine (SMM) throughout the kiln-drying of the malt and the hot stages of the brewing process (wort boiling and clarification), or during fermentation by the yeast reduction of dimethylsulfoxide (DMSO) [54]. Additionally, when the content of DMSO in the wort at pitching is high, the concentration of DMS in the beer will be high as well [55]. When present at high concentrations, DMS gives an unpleasant taste and has the aroma of cooked, sweet corn [36]. However, in lager beers and at moderate levels (30–100 ppb), DMS is considered an essential component [56].

### 3.3. Organoleptic Test

**Figure 5:**
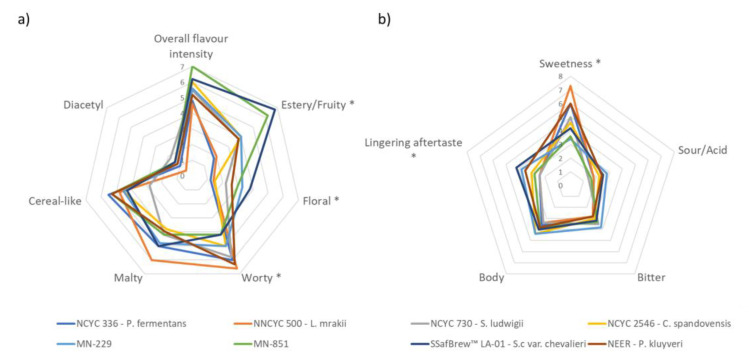
Sensory analysis of the eight beers studiedL (**a**) aroma attributes: overall flavor intensity, estery/fruity, floral, worty, malty, cereal-like, diacetyl. (**b**) Taste attributes: sweetness, sour/cid, bitter, body, and lingering aftertaste. * Significantly different (ANOVA; *p* < 0.05).

Overall, the studied beers presented volatile compounds in concentrations below traditional beer thresholds. Nevertheless, several of these volatile compounds could still be perceived. The perception threshold value for a given compound is often affected by the content of the other compounds in the beverage matrix. Apart from the volatile compounds’ synergistic effects in detection, the NABLABs´ low-ethanol and high-sugar concentrations are also important. A previous study demonstrated that the volatile compounds 2-methylbutanal and 3-methylbutanal showed increased retention when the ethanol concentration in an aqueous solution was increased from 0 to 5% [58]. This observation was explained by the cosolvent effect of ethanol in water, which increased the solubility of these volatile compounds, leading to a reduction in their partition coefficient between the solution and air [59]. On the other hand, an increased concentration of sugar increases the release of aromatic compounds. A previous study showed that the content of sugars in beer led to an increase in the release of 2- and 3-methylbutanal, up to a maximum sugar concentration of 40 g/L [58]. Figure 5 represents a spider graph employing key descriptors considered in the sensory tests for the eight beers obtained in the 2 L fermentations. At the end of the experience, LA-01 *S. cerevisiae* showed significantly intense estery/fruity characteristics, followed by MN-851, also with significantly intense estery/fruity scores, while the beers produced by the other yeasts were poorly pronounced in this descriptor. Regarding a floral aroma, the beers produced by LA-01 *S. cerevisae* showed a significantly pronounced score when compared to the beer produced by *P. fermentans*, *L. mrakii*, and *C. spandovensis*. The beer produced by MN-229 showed a significant difference when compared with *P. fermentans*.

The beers produced by *P. fermentans, L. mrakii, S. ludwigii*, and Neer *P. kluyveri*, mainly showed high aroma intensity values for the worty, malty, and cereal-like attributes (Table 3). Concerning the sweet flavor, the beer produced by *P. fermentans* was significantly sweeter than the beerss produced by MN-229 and MN-851. Furthermore, the *L. mrakii* beer showed a sweet flavor profile significantly more pronounced than MN-229, MN-851, *S. ludwigii*, *C. spandovensis*, and LA-01 *S. cerevisiae*, beers that demonstrated higher ethanol concentrations and less residual sugars. However, all tested beers showed high concentration values for maltose (Table 3), leading to a thick mouthfeel and mildly sweet flavor. This higher sweetness and mouthfeel have been reported previously in yeasts with a low capacity to ferment maltose [7]. The lingering aftertaste was more marked in LA-01 *S. cerevisiae* when compared to *P. fermentans*, *L. mrakii*, and *S. ludwigii*. Furthermore, tasters indicated the *L. mrakii* beer as having a glue-like off-flavor, which can be connected to its higher content of ethyl acetate, as well as a cooked vegetable flavor, which can be linked to its higher DMS content in DMS (Table 4). Wort with less available maltose should positively impact the sensory output. 

**Figure 6:**
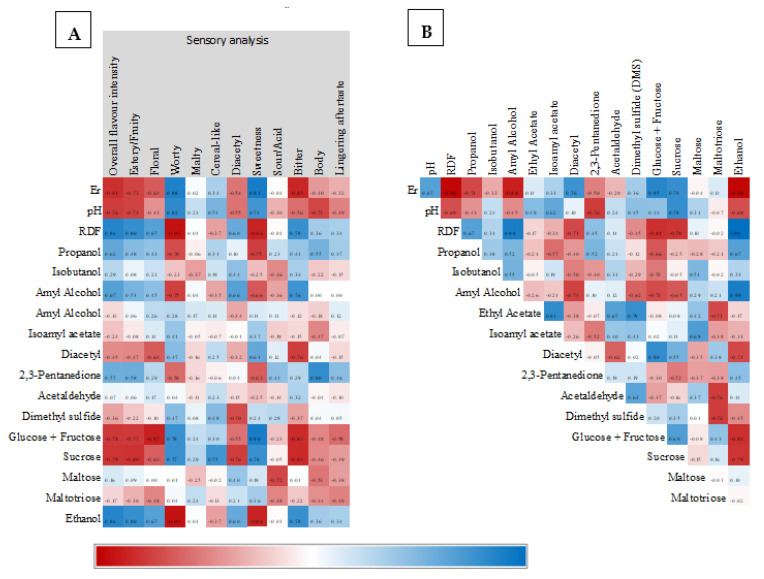
Heat map of Spearman’s values for each characteristic measure in produced beers. Sensory analysis aroma attributes are shaded in gray. (**A**) Sensory analysis vs beer attributes and (**B**) Beer attributes vs beer attributes.

With the objective of better understanding the connection between different beer attributes and sensory analyses, a Spearman correlation test was applied to the previously quantified data (Figure 6). Overall flavor intensity positively correlates and is statistically significant (Table S1) with RDF, ethanol content, and the estery/fruity and floral attributes. On the other hand, there is a negative correlation with statistical significance and the pH, ER, sucrose, glucose, and fructose, implying that high sugar and high pH values were not appreciated by the taster panel. Each beer and its characteristics were also submitted to PCA (Figure S2), and it was possible to observe that beers that showed more acceptance LA-01 *S. cerevisiae*, MN-229 and MN-851, were grouped together. Characteristics such as isoamyl acetate, ethyl acetate, sucrose, glucose, and fructose concentrations explain the major divergence between beers in the PCA analysis.

## 4. Conclusions

Currently, there is an increasing customer demand for diversity in beer styles, thus stimulating the search for new approaches, including the use of alternative yeasts. From the initial ten yeasts tested, *H. valbyensis* and *Z. rouxii* were able to ferment maltose, revealing that they are unsuitable to produce NABLABs using a biological approach. However, these yeasts could prove to be interesting for the production of NABLABs through de-alcoholization. *L. mrakii* demonstrated a high ester production but had no positive results to produce beer as a single-fermentation yeast. However, this species could have the potential to produce beer through a co-culture approach. *L. mrakii* and Neer *P. kluvery* produced beers with an ethanol content below 0.5% (*v/v*), making them eligible for the production of non-alcoholic beers. The remaining strains produced beers with an ethanol content below 1.2% (*v/v*), making them eligible for the production of low-alcoholic beers. Worty, malty, cereal-like, and sweet were the biggest defects pointed by the tasting panel.

The yeasts that showed a higher potential for lager beer production and acceptance by the taste panelists were the *S. cerevisiae*-derived strains: MN-229, MN-851, and *Saccharomyces* LA-01. Furthermore, these strains demonstrated volatile compounds at desirable concentrations. These results highlight the potential of yeast selection, sexual reproduction, and hybridization for the attainment of novel beer characteristics. Non-traditional beer yeasts proved to be suitable for producing a NABLAB by a biological approach or to produce novel, non-traditional beers. 

## Figures and Tables

**Table 1:** Yeast strain designation, species, and origin of yeast strains used in this study. MN-229 and MN-851, are new yeast strains.

Strain Designation	Species	Origin	Yeast Bank
NCYC 328	*Hanseniaspora valbyensis*	Draught beer, UK.	NCYC, National Collection of Yeast Cultures
NCYC 336	*Pichia fermentans*	Draught beer, UK.	NCYC, National Collection of Yeast Cultures
NCYC 500	*Lindnera mrakii*	Soil, Dobodura area, Papua New Guinea	NCYC, National Collection of Yeast Cultures
NCYC 730	*Saccharomycodes ludwigii*	Isolated from grape must, Germany	NCYC, National Collection of Yeast Cultures
NCYC 1508	*Zygosaccharomyces rouxii*	Isolated from 70% malt extract	NCYC, National Collection of Yeast Cultures
NCYC 2546	*Candida spandovensis*	Beer, Germany	NCYC, National Collection of Yeast Cultures
MN-229	*Saccharomyces cerevisiae*	Engineered organism	Renaissance BioScience
MN-851	*Saccharomyces cerevisiae*	Engineered organism	Renaissance BioScience
SafBrew™ LA-01	*Saccharomyces cerevisiae var. Chevalieri*	Selected from Saf Colection	Fermentis
NEER	*Pichia kluyveri*	Isolated in New Zealand	Chr. Hansen SmartBevTM

**Table 2:** Concentration of carbohydrates, glycerol, and ethanol in Biolector assays, expressed as the mean concentration with standard deviation.

Strain Designation	Ethanol (g L ⁻¹)			Glycerol (g L ⁻¹)			Maltose (g L ⁻¹)			Fructose (g L ⁻¹)			Glucose (g L ⁻¹)		
Wort		n.d.			n.d.		65.52	±	0.97	1.85	±	0.11	11.68	±	0.20
NCYC 328—*H. valbyensis*	26.29	±	4.56	1.24	±	0.33		n.d.		0.50	±	0.51	0.36	±	0.41
NCYC 336—*P. fermentans*	0.40	±	0.56		n.d.		66.90	±	1.51		n.d.			n.d.	
NCYC 500—*L. mrakii*		n.d.			n.d.		62.83	±	5.73		n.d.			n.d.	
NCYC 730—*S. ludwigii*		n.d.			n.d.		63.87	±	5.27	0.29	±	0.04		n.d.	
NCYC 1508 - *Z. rouxii*	7.12	±	6.44	2.21	±	0.88	12.65	±	8.05	0.16	±	0.23	2.00	±	0.80
NCYC 2546—*C. spandovensis*	0.46	±	0.64	0.14	±	0.20	59.76	±	6.21		n.d.			n.d.	
MN 229		n.d.			n.d.		64.47	±	3.13		n.d.			n.d.	
MN 851		n.d.			n.d.		68.61	±	1.76	0.24	±	0.03		n.d.	
SafBrew™ LA-01—*S.c var. chevalieri*		n.d.			n.d.		62.07	±	0.62		n.d.			n.d.	
NEER—*P. kluyveri*		n.d.			n.d.		65.46	±	3.68		n.d.			n.d.	

n.d.—not detected.

**Table 3:** Concentration of carbohydrates and ethanol in green beer, expressed as mean concentration of analysis duplicate, including standard deviation.

Strain Designation	Ethanol			Maltotriose			Maltose			Sucrose			Glucose + Fructose		
	(%*v/v*)			(g L ⁻¹)			(g L ⁻¹)			(g L ⁻¹)			(g L ⁻¹)		
Wort		n.d.		16.60	±	0.10	56.50	±	1.30	2.20	±	0.40	15.95	±	0.05
NCYC 336—*P. fermentans*	0.52	±	0.03	16.35	±	0.45	53.60	±	0.40	2.55	±	0.05	8.10	±	0.60
NCYC 500—*L. mrakii*	0.30	±	0.12	15.30	±	0.30	54.15	±	0.75	1.75	±	0.05	8.25	±	0.11
NCYC 730—*S. ludwigii*	0.92	±	0.05	16.05	±	0.05	54.25	±	0.25		n.d.		4.00	±	0.80
NCYC 2546—*C. spandovensis*	0.66	±	0.27	15.95	±	0.15	53.85	±	0.25		n.d.		7.70	±	4.50
MN-229	1.17	±	0.04	14.60	±	0.20	52.55	±	0.35		n.d.			n.d.	
MN-851	1.19	±	0.01	15.20	±	0.30	55.25	±	1.25		n.d.			n.d.	
SafBrew™ LA-01 - *S.c var. chevalieri*	1.19	±	0.06	16.00	±	0.10	53.85	±	1.05		n.d.		1.00	±	0.00
NEER—*P. kluyveri*	0.17	±	0.03	15.05	±	0.05	54.15	±	0.25	1.90	±	0.00	7.35	±	0.25

n.d.—not detected.

**Table 4:** Characterization of main volatile compounds quantified from beer produced in 2 L fermentation with selected yeasts. Values correspond to the mean and standard deviation. Detection threshold values refer to values obtained from traditional alcoholic beer.

Compound	Odor Description	Detection Threshold (mg L ⁻¹)	NCYC 336—*P. fermentans*			NCYC 500—*L. mrakii*			NCYC 730—*S. ludwigii*			NCYC 2546—*C. spandovensis*			NEER—*P. kluyveri*			SafBrew™ LA-01			MN-229			MN-851			
			(mg L ⁻¹)			(mg L ⁻¹)			(mg L ⁻¹)			(mg L ⁻¹)			(mg L ⁻¹)			(mg L ⁻¹)			(mg L ⁻¹)			(mg L ⁻¹)			
**Higher alcohols**																											
Propanol	Alcohol ^a^	600 ^e^, 800 ^f,g^	4.40	±	3.45	2.34	±	0.04	3.06	±	0.12	3.96	±	1.72	3.15	±	0.55	4.21	±	0.63	5.46	±	0.61	9.90	±	0.19	
Isobutanol	Alcohol ^a^	100 ^e^, 200 ^f,g^	5.87	±	0.41	3.16	±	0.06	9.55	±	0.03	3.81	±	1.34	7.22	±	1.94	4.40	±	0.07	4.88	±	0.94	11.32	±	0.06	
Amyl Alcohol	Alcohol, banana, medicinal, solvent, fruity ^a^	50–70 ^h^	9.46	±	0.94	5.69	±	0.06	20.87	±	0.31	8.07	±	3.24	5.06	±	1.41	20.45	±	0.60	15.73	±	3.17	36.06	±	0.17	
**Esters**																											
Ethyl Acetate	Solvent, fruity, sweetish ^b^	25–30 ^h^	0.67	±	0.13	52.18	±	25.54	2.27	±	0.19	1.59	±	1.01	32.59	±	8.27	2.79	±	0.03	3.65	±	0.87	5.56	±	0.26	
Isoamyl acetate	Banana, apple, solvent, estery, pear ^b^	1.2 ^f,h,i,^2 ^h^	0.00	±	0.00	0.94	±	0.24	0.83	±	0.30	0.26	±	0.24	9.06	±	1.94	0.38	±	0.02	0.35	±	0.04	0.69	±	0.01	
**Vicinal diketones**																											
Diacetyl	Butter ^a^	0.15 ^c^	0.14	±	0.02	0.11	±	0.00	0.07	±	0.01	0.31	±	0.03	0.11	±	0.03	0.08	±	0.01	0.06	±	0.02	0.04	±	0.00	
2,3-Pentanedione	Honey, toffee-like ^c^	1–1.5 ^j^	0.02	±	0.00	0.02	±	0.00	0.02	±	0.00	0.08	±	0.04	0.02	±	0.00	0.03	±	0.01	0.04	±	0.01	0.03	±	0.00	
**Aldehydes**																											
Acetaldehyde	Grassy, green leaves, fruity ^a^	10–25 ^k^	10.80	±	2.91	20.10	±	3.54	13.29	±	3.18	12.34	±	10.19	15.45	±	3.35	1.47	±	0.05	25.83	±	20.78	30.18	±	20.53	
**Sulfur compounds**																											
Dimethyl sulfide (DMS)	Cabbagy, Cooked-vegetable ^d^	0.025—0.030 ^d^, 0.05 ^i^	9.15	±	0.35	20.30	±	0.00	6.85	±	1.35	9.45	±	1.15	16.55	±	3.45	8.30	±	0.50	16.50	±	1.50	11.95	±	3.85	

a [33], b [57], c [27], d [51], e [28], f [24], g [29], h [30], i [37], j [44], and k [46].

## Data Availability

Not applicable.

## Supplementary Materials

The following supporting information can be downloaded at: https://www.mdpi.com/article/10.3390/microorganisms11020316/s1, Figure S1. pH was measured for 10–12 days, as reported for fermentations of all yeast in this study. Results are reported as the mean of two biological replicates. Figure S2. Scatter Plot analysis PCA. PC1(52.3%) vs PC2(20.2%), Table S1. Spearman’s P-values, highlighted at pink P-values below 0.05.
