# Supplementary material for: Exploiting Non-Conventional Yeasts for Low-Alcohol Beer Production
Source: Microorganisms. 2023 Jan 26;11(2):316. doi: 10.3390/microorganisms11020316 (PMC9961705; doi:10.3390/microorganisms11020316)
Supplement: Supplementary file 1 [file microorganisms-11-00316-s001.zip › microorganisms-2156330-supplementary.pdf]

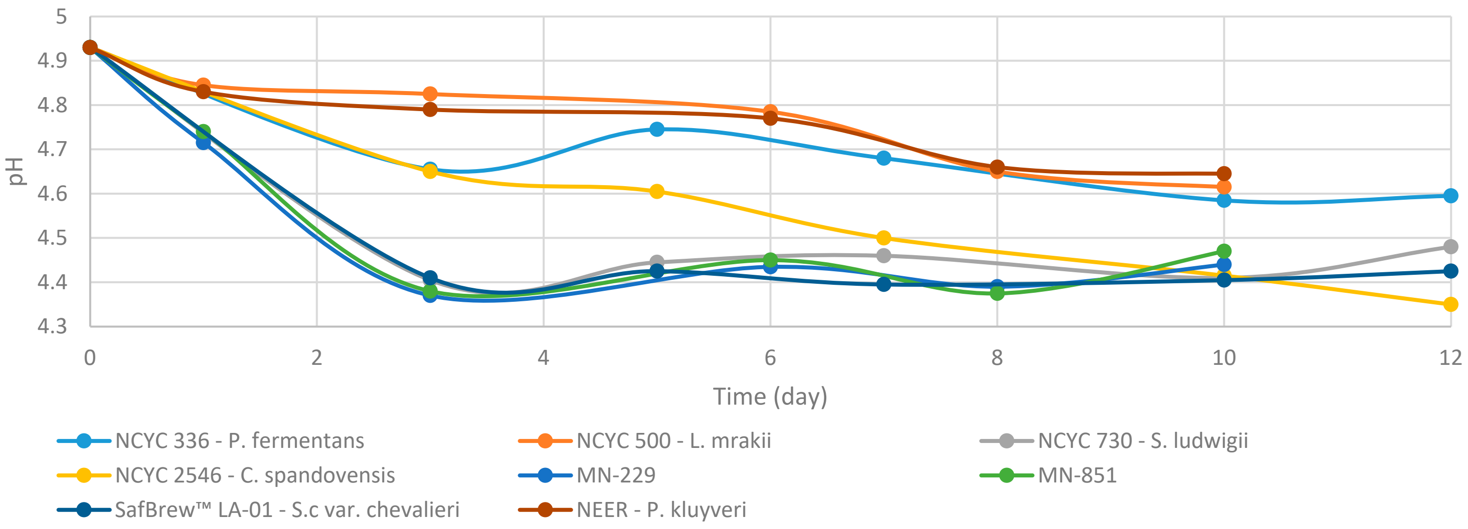

Figure S1. pH was measured for 10-12 days, as reported for fermentations of all yeast in this study. Results are reported as the mean of two biological replicates.

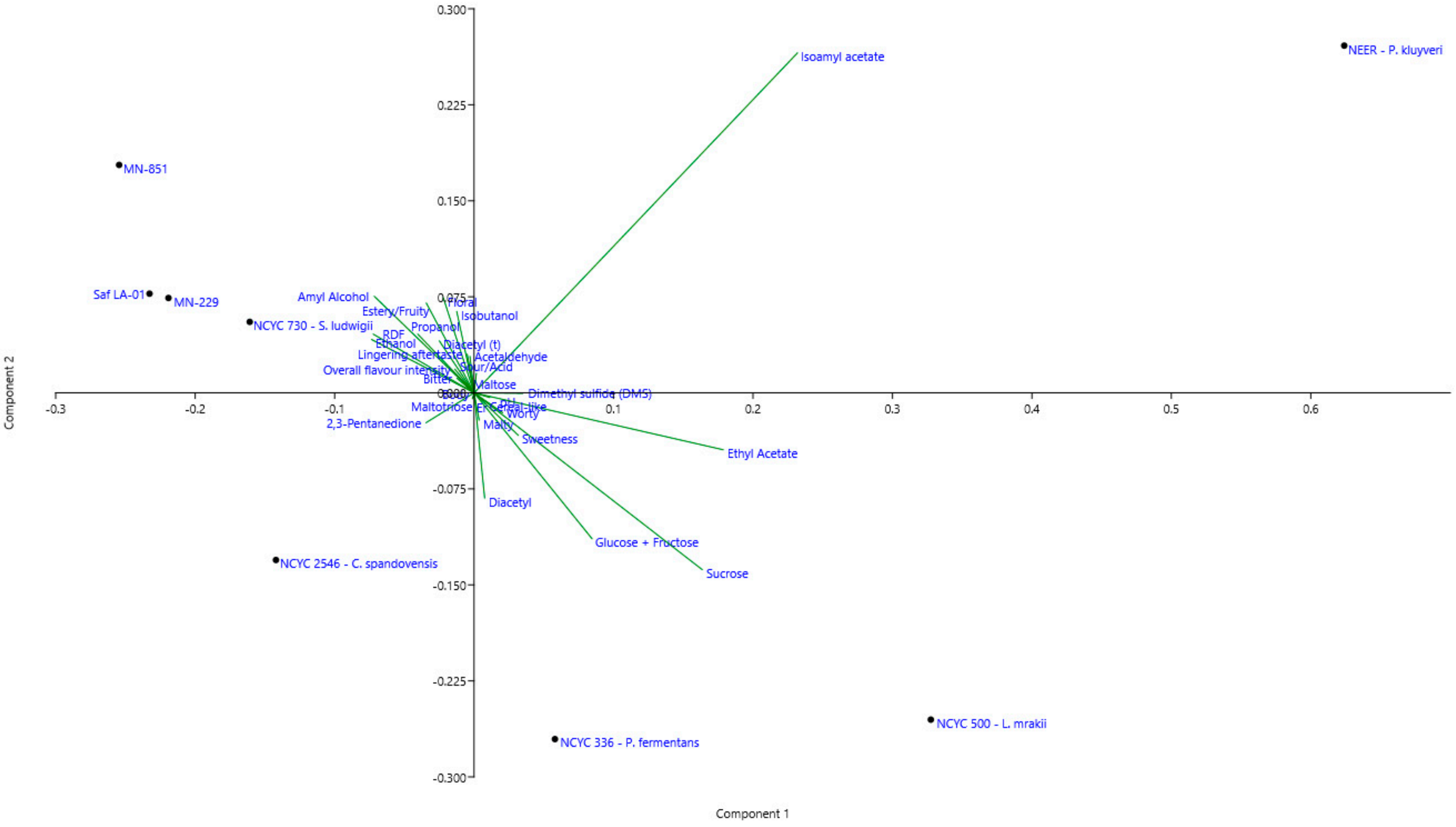

Figure S2. Scatter Plot analysis PCA. PC1(52.3%) vs PC2(20.2%).

Table S1. Spearman's P-values, highlighted at pink *P*-values below 0.05.

																			Sensory analysis											
		Er (%m/m)	pH	RDF	Propanol	Isobutanol	Amyl Alcohol	Ethyl Acetate	Isoamyl acetate	Diacetyl	2,3-Pentanedione	Acetaldehyde	Dimethyl sulfide (DMS)	Glucose + Fructose	Sucrose	Maltose	Maltotriose	Ethanol	Overall flavour intensity	Estery/Fruity	Floral	Worty	Malty	Cereal-like	Diacetyl (t)	Sweetness	Sour/Acid	Bitter	Body	Lingering aftertaste
	Er (%m/m)		0.083	0.000	0.058	0.428	0.007	0.752	0.428	0.037	0.216	0.501	0.389	0.012	0.036	0.941	0.752	0.000	0.022	0.045	0.096	0.006	0.967	0.417	0.171	0.003	0.990	0.013	0.343	0.449
	pH	0.083		0.069	0.299	0.582	0.267	0.243	0.115	0.840	0.037	0.582	0.267	0.273	0.036	0.448	0.882	0.069	0.037	0.045	0.299	0.019	0.587	0.182	0.160	0.042	0.471	0.161	0.055	0.227
	RDF	0.000	0.069		0.083	0.462	0.005	0.703	0.462	0.058	0.267	0.752	0.267	0.019	0.036	0.829	0.977	0.000	0.011	0.023	0.083	0.002	0.988	0.363	0.120	0.009	0.990	0.032	0.384	0.410
	Propanol	0.058	0.299	0.083		0.360	0.197	0.582	0.151	0.327	0.197	0.619	0.793	0.085	0.560	0.507	0.619	0.083	0.115	0.232	0.299	0.063	0.895	0.448	0.826	0.038	0.589	0.313	0.183	0.379
	Isobutanol	0.428	0.582	0.462	0.360		0.171	0.935	0.665	0.216	0.327	0.462	0.501	0.185	0.911	0.206	0.977	0.462	0.501	0.850	0.619	0.585	0.363	0.654	0.277	0.550	0.260	0.410	0.615	0.743
	Amyl Alcohol	0.007	0.267	0.005	0.197	0.171		0.536	0.619	0.046	0.840	0.793	0.115	0.060	0.089	0.487	0.619	0.005	0.083	0.184	0.267	0.041	0.940	0.389	0.079	0.084	0.381	0.041	1.000	1.000
	Ethyl Acetate	0.752	0.243	0.703	0.582	0.935	0.536		0.022	0.360	0.882	0.083	0.028	0.849	0.875	0.299	0.058	0.703	0.752	0.891	0.536	0.504	0.692	0.736	0.404	0.736	0.808	0.794	0.692	0.794
	Isoamyl acetate	0.428	0.115	0.462	0.151	0.665	0.619	0.022		0.536	0.197	0.327	0.299	0.964	0.827	0.067	0.360	0.462	0.582	0.850	0.752	0.313	0.924	0.871	0.990	0.363	0.646	0.743	0.255	0.887
	Diacetyl	0.037	0.840	0.058	0.327	0.216	0.046	0.360	0.536		0.935	0.115	0.977	0.022	0.167	0.396	0.360	0.058	0.267	0.245	0.096	0.244	0.716	0.541	0.439	0.117	0.781	0.041	0.971	0.743
	2,3-Pentanedione	0.216	0.037	0.267	0.197	0.327	0.840	0.882	0.197	0.935		0.665	0.665	0.468	0.208	0.364	0.360	0.267	0.151	0.141	0.501	0.141	0.716	0.895	0.943	0.100	0.275	0.483	0.025	0.255
	Acetaldehyde	0.501	0.582	0.752	0.619	0.462	0.793	0.083	0.327	0.115	0.665		0.096	0.363	0.702	0.364	0.037	0.752	0.882	0.891	0.703	0.942	0.801	0.587	0.729	0.550	0.735	0.449	0.971	0.830
	Dimethyl sulfide (DMS)	0.389	0.267	0.267	0.793	0.501	0.115	0.028	0.299	0.977	0.665	0.096		0.629	0.399	0.989	0.037	0.267	0.389	0.603	0.840	0.244	0.852	0.233	0.062	0.566	0.507	0.379	0.971	0.928
	Glucose + Fructose	0.012	0.273	0.019	0.085	0.185	0.060	0.849	0.964	0.022	0.468	0.363	0.629		0.074	0.841	0.285	0.019	0.030	0.030	0.008	0.031	0.570	0.459	0.160	0.004	0.579	0.016	0.238	0.141
	Sucrose	0.036	0.036	0.036	0.560	0.911	0.089	0.875	0.827	0.167	0.208	0.702	0.399	0.074		0.762	0.702	0.036	0.036	0.036	0.113	0.048	0.494	0.048	0.042	0.036	0.905	0.027	0.286	0.348
	Maltose	0.941	0.448	0.829	0.507	0.206	0.487	0.299	0.067	0.396	0.364	0.364	0.989	0.841	0.762		0.989	0.829	0.711	0.850	1.000	0.982	0.540	0.965	0.320	0.675	0.049	0.989	0.183	0.343
	Maltotriose	0.752	0.882	0.977	0.619	0.977	0.619	0.058	0.360	0.360	0.360	0.037	0.037	0.285	0.702	0.989		0.977	0.703	0.464	0.243	0.942	0.564	0.756	0.623	0.379	0.233	0.617	0.290	0.227
	Ethanol	0.000	0.069	0.000	0.083	0.462	0.005	0.703	0.462	0.058	0.267	0.752	0.267	0.019	0.036	0.829	0.977		0.011	0.023	0.083	0.002	0.988	0.363	0.120	0.009	0.990	0.032	0.384	0.410
Sensory analysis	Overall flavour intensity	0.022	0.037	0.011	0.115	0.501	0.083	0.752	0.582	0.267	0.151	0.882	0.389	0.030	0.036	0.711	0.703	0.011		0.001	0.058	0.001	0.329	0.448	0.054	0.011	0.761	0.122	0.123	0.161
	Estery/Fruity	0.045	0.045	0.023	0.232	0.850	0.184	0.891	0.850	0.245	0.141	0.891	0.603	0.030	0.036	0.850	0.464	0.023	0.001		0.010	0.004	0.530	0.283	0.114	0.017	0.390	0.139	0.084	0.042
	Floral	0.096	0.299	0.083	0.299	0.619	0.267	0.536	0.752	0.096	0.501	0.703	0.840	0.008	0.113	1.000	0.243	0.083	0.058	0.010		0.063	0.801	0.298	0.277	0.033	0.197	0.104	0.183	0.017
	Worty	0.006	0.019	0.002	0.063	0.585	0.041	0.504	0.313	0.244	0.141	0.942	0.244	0.031	0.048	0.982	0.942	0.002	0.001	0.004	0.063		0.615	0.409	0.088	0.006	0.680	0.091	0.125	0.166
	Malty	0.967	0.587	0.988	0.895	0.363	0.940	0.692	0.924	0.716	0.716	0.801	0.852	0.570	0.494	0.540	0.564	0.988	0.329	0.530	0.801	0.615		0.859	0.149	0.551	0.928	0.683	0.324	0.648
	Cereal-like	0.417	0.182	0.363	0.448	0.654	0.389	0.736	0.871	0.541	0.895	0.587	0.233	0.459	0.048	0.965	0.756	0.363	0.448	0.283	0.298	0.409	0.859		0.090	0.415	0.819	0.112	0.727	0.515
	Diacetyl (t)	0.171	0.160	0.120	0.826	0.277	0.079	0.404	0.990	0.439	0.943	0.729	0.062	0.160	0.042	0.320	0.623	0.120	0.054	0.114	0.277	0.088	0.149	0.090		0.197	0.532	0.104	0.665	0.656
	Sweetness	0.003	0.042	0.009	0.038	0.550	0.084	0.736	0.363	0.117	0.100	0.550	0.566	0.004	0.036	0.675	0.379	0.009	0.011	0.017	0.033	0.006	0.551	0.415	0.197		0.399	0.016	0.058	0.115
	Sour/Acid	0.990	0.471	0.990	0.589	0.260	0.381	0.808	0.646	0.781	0.275	0.735	0.507	0.579	0.905	0.049	0.233	0.990	0.761	0.390	0.197	0.680	0.928	0.819	0.532	0.399		0.922	0.040	0.010
	Bitter	0.013	0.161	0.032	0.313	0.410	0.041	0.794	0.743	0.041	0.483	0.449	0.379	0.016	0.027	0.989	0.617	0.032	0.122	0.139	0.104	0.091	0.683	0.112	0.104	0.016	0.922		0.423	0.483
	Body	0.343	0.055	0.384	0.183	0.615	1.000	0.692	0.255	0.971	0.025	0.971	0.971	0.238	0.286	0.183	0.290	0.384	0.123	0.084	0.183	0.125	0.324	0.727	0.665	0.058	0.040	0.423		0.021
	Lingering aftertaste	0.449	0.227	0.410	0.379	0.743	1.000	0.794	0.887	0.743	0.255	0.830	0.928	0.141	0.348	0.343	0.227	0.410	0.161	0.042	0.017	0.166	0.648	0.515	0.656	0.115	0.010	0.483	0.021	
